# Geographic variation in fitness‐related traits of the bladderwrack *Fucus vesiculosus* along the Baltic Sea‐North Sea salinity gradient

**DOI:** 10.1002/ece3.5470

**Published:** 2019-07-23

**Authors:** Francisco R. Barboza, Jonne Kotta, Florian Weinberger, Veijo Jormalainen, Patrik Kraufvelin, Markus Molis, Hendrik Schubert, Henrik Pavia, Göran M. Nylund, Lena Kautsky, Ellen Schagerström, Esther Rickert, Mahasweta Saha, Stein Fredriksen, Georg Martin, Kaire Torn, Ari Ruuskanen, Martin Wahl

**Affiliations:** ^1^ GEOMAR Helmholtz Centre for Ocean Research Kiel Kiel Germany; ^2^ Estonian Marine Institute University of Tartu Tallinn Estonia; ^3^ Department of Biology University of Turku Turku Finland; ^4^ Department of Aquatic Resources, Institute of Coastal Research Swedish University of Agricultural Sciences Öregrund Sweden; ^5^ Alfred‐Wegener‐Institute Helmholtz Centre for Polar and Marine Research Bremerhaven Germany; ^6^ Biosciences, University Rostock Rostock Germany; ^7^ Department of Marine Sciences – Tjärnö University of Gothenburg Strömstad Sweden; ^8^ Baltic Sea Centre Stockholm University Stockholm Sweden; ^9^ Department of Ecology, Environment & Plant Sciences Stockholm University Stockholm Sweden; ^10^ School of Biological Sciences University of Essex Colchester UK; ^11^ Marine Ecology and Biodiversity Plymouth Marine Laboratory Plymouth UK; ^12^ Department of Biosciences University of Oslo Oslo Norway; ^13^ Tvärminne Zoological Station Hanko Finland

**Keywords:** environmental gradient, foundation species, *Fucus vesiculosus*, intraspecific variation

## Abstract

In the course of the ongoing global intensification and diversification of human pressures, the study of variation patterns of biological traits along environmental gradients can provide relevant information on the performance of species under shifting conditions. The pronounced salinity gradient, co‐occurrence of multiple stressors, and accelerated rates of change make the Baltic Sea and its transition to North Sea a suitable region for this type of study. Focusing on the bladderwrack *Fucus vesiculosus*, one of the main foundation species on hard‐bottoms of the Baltic Sea, we analyzed the phenotypic variation among populations occurring along 2,000 km of coasts subjected to salinities from 4 to >30 and a variety of other stressors. Morphological and biochemical traits, including palatability for grazers, were recorded at 20 stations along the Baltic Sea and four stations in the North Sea. We evaluated in a common modeling framework the relative contribution of multiple environmental drivers to the observed trait patterns. Salinity was the main and, in some cases, the only environmental driver of the geographic trait variation in *F. vesiculosus*. The decrease in salinity from North Sea to Baltic Sea stations was accompanied by a decline in thallus size, photosynthetic pigments, and energy storage compounds, and affected the interaction of the alga with herbivores and epibiota. For some traits, drivers that vary locally such as wave exposure, light availability or nutrient enrichment were also important. The strong genetic population structure in this macroalgae might play a role in the generation and maintenance of phenotypic patterns across geographic scales. In light of our results, the desalination process projected for the Baltic Sea could have detrimental impacts on *F. vesiculosus* in areas close to its tolerance limit, affecting ecosystem functions such as habitat formation, primary production, and food supply.

## 1 INTRODUCTION

In the course of global change, the shift in environmental conditions is faster than ever and the distributional ranges of species are or will be on the move (Nicastro et al., 2013; Wernberg et al., 2011)—unless populations can adapt fast enough. In this context, marginal seas (e.g., Baltic Sea and Mediterranean Sea) may represent natural laboratories where to study the biological consequences of future environmental scenarios because (a) changes are faster in these smaller and more land‐locked water bodies than in the open sea and (b) species live under conditions close to their tolerance limits regarding various environmental factors (e.g., Meier et al., 2012; Reusch et al., 2018; Rilov, 2016; Schroeder et al., 2017). Complementarily, studies on the trait variation of populations along environmental gradients are helpful in revealing the capacity to respond to environmental changes that species have across (or beyond) their distribution. For macroalgae, the subject of the present study, several such efforts have been undertaken along latitudinal gradients (Lima, Ribeiro, Queiroz, Hawkins, & Santos, 2007; Nicastro et al., 2013; Wernberg et al., 2011; Zardi et al., 2015) and predominantly regarding sea surface temperature as a potential stressor. These studies have provided valuable knowledge on interpopulation differences in the tolerance to temperature and desiccation, or resistance to pathogens. The Baltic Sea is particularly suited for this kind of studies given (a) the pronounced salinity gradient from over 30 to below 2 along 2,000 km; (b) the spatial heterogeneity in the (co‐)occurrence of several stressors such as warming, acidification, eutrophication, desalination, and deoxygenation; and (c) the unusually fast rate of past and ongoing environmental changes (Meier et al., 2012; Reusch et al., 2018).

Habitat‐forming macroalgae are under severe environmental pressures worldwide (Wahl et al., 2015). One of these, the bladderwrack *Fucus vesiculosus* (Linnaeus 1753, Figure 1), is distributed across the North Atlantic Ocean and is the major canopy‐forming species on hard‐bottoms of the Baltic Sea (Wikström & Kautsky, 2007), where it occurs along most of the salinity gradient of this region (Schubert, Feuerpfeil, Marquardt, Telesh, & Skarlato, 2011). Regional patterns in traits and performance of *F. vesiculosu*s have previously been investigated along its latitudinal range in the North‐East Atlantic (e.g., Assis, Serrao, Claro, Perrin, & Pearson, 2014; Mota, Engelen, Serrao, & Pearson, 2015; Nicastro et al., 2013; Pearson, Lago‐Leston, & Mota, 2009). At its southern margin, which is moving northwards due to increasing heat stress (Nicastro et al., 2013), *F. vesiculosus* features lower reproductive success and recruitment as compared to core habitats (Ferreira, Hawkins, & Jenkins, 2015), but no changes in heat‐shock response have been observed (Pearson et al., 2009). Interestingly, at these southern and thermally more stressful regions, this marine alga predominantly occurs in brackish environments (Mota et al., 2015). Although *F. vesiculosus* has a wide salinity tolerance (from 32 to 4), given the physiological significance of salinity for aquatic organisms, it should be expected that either the performance of populations declines from marine to brackish conditions along the Baltic Sea or that they are locally adapted. Previous comparisons of single populations from contrasting salinity conditions have described shorter thalli, smaller growth rates, and less active photosynthetic systems in individuals from a brackish station of the northern Baltic Sea than in individuals from a fully marine station of the Irish Sea (Bäck, Collins, & Russell, 1992a, 1992b; Nygård & Dring, 2008). When cultivated at different salinities, individuals from both populations performed better in fully marine conditions, but Baltic *F. vesiculosus* individuals were able to grow at lower salinities than the Irish ones (Nygård & Dring, 2008). Although there is limited evidence to state that observed interpopulation differences in response to salinity changes are the result of genetic differentiation and local adaptation, these processes could be favored in *F. vesiculosus* due to the limited gene flow caused by its extremely short‐range gamete dispersal, which rarely exceeds few meters (Tatarenkov, Jönsson, Kautsky, & Johannesson, 2007). In addition to the potential genetic differentiation, phenotypic plasticity may contribute to the observed variation of traits and affect the capacity of the species to cope with environmental changes (Kawecki & Ebert, 2004; Reusch & Wood, 2007).

**Figure 1:**
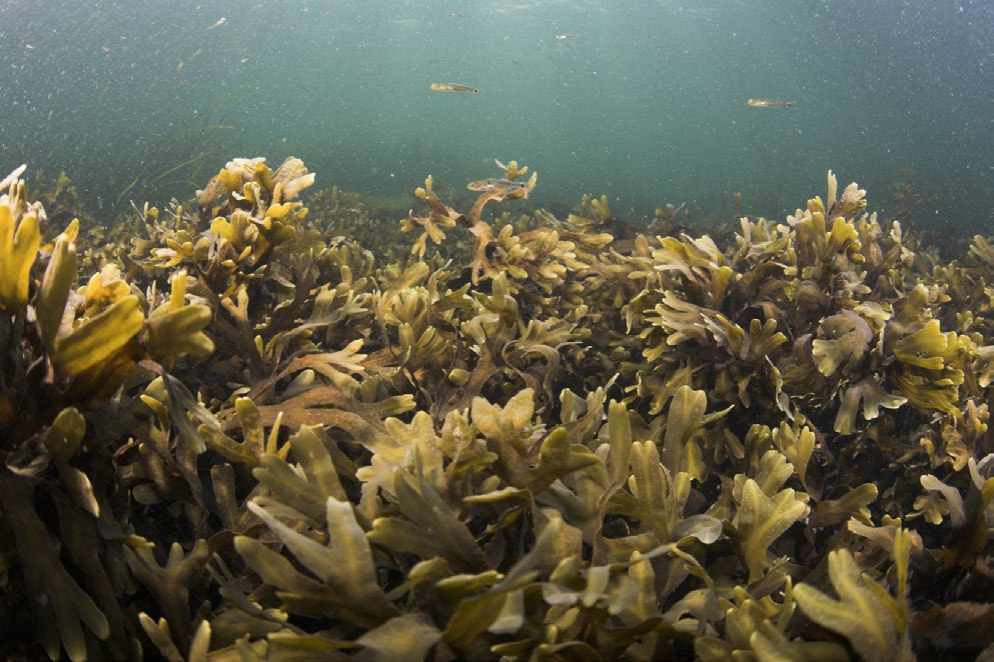
A stand of the bladderwrack *Fucus vesiculosus* in the southwestern Baltic Sea (photo by: Uli Kunz)

In a Baltic‐wide assessment, we investigated the variation of multiple traits including morphology, light‐harvesting pigments, chemical composition, and interactions of *F. vesiculosus* with consumers and epibiota along the Baltic Sea‐North Sea salinity gradient. Despite the overwhelming influence of salinity on marine diversity (e.g., Schubert et al., 2011) and the expected desalination in the Baltic Sea (Meier, Kjellström, & Graham, 2006), the geographic variation of *F. vesiculosus*' traits has not yet been thoroughly documented in this region. While salinity constitutes the most prominent environmental gradient in the Baltic, other factors such as nutrient availability, irradiance, or wave exposure may also affect the performance of *F. vesiculosus* at sub‐regional and local scales. Sampling populations from 20 stations of the Baltic Sea and four stations from the North Sea, we assessed the geographic changes in the fitness and ecological performance of *F. vesiculosus* by correlating salinity and other environmental variables to thallus size, content of photosynthetic pigments and energy storage compounds, and susceptibility to natural enemies (considered here as fitness proxies). Numerous studies described negative direct and indirect effects of decreasing salinity, nutrient enrichment and increasing wave exposure on the morphology, growth, photosynthesis, palatability, and epibiosis—among others—of *F. vesiculosus* (e.g., Bergström, Berger, & Kautsky, 2003; Hemmi, Mäkinen, Jormalainen, & Honkanen, 2005; Kalvas & Kautsky, 1993; Kersen, Kotta, Bučas, Kolesova, & Deķere, 2011; Takolander, Leskinen, & Cabeza, 2017). In light of this evidence, we expected that the effects of the Baltic Sea–North Sea salinity gradient together with other environmental variables of local relevance would result in (a) biochemical and morphological differences among *F. vesiculosus* populations, and (b) an overall decrease in the performance of the species with decreasing salinity. The effort to relate this variation with environmental conditions within the sampled habitats may provide new insights into the capacity of *F. vesiculosus* to respond to future changes.

## 2 MATERIALS AND METHODS

### 2.1 Collection of algae

**Figure 2:**
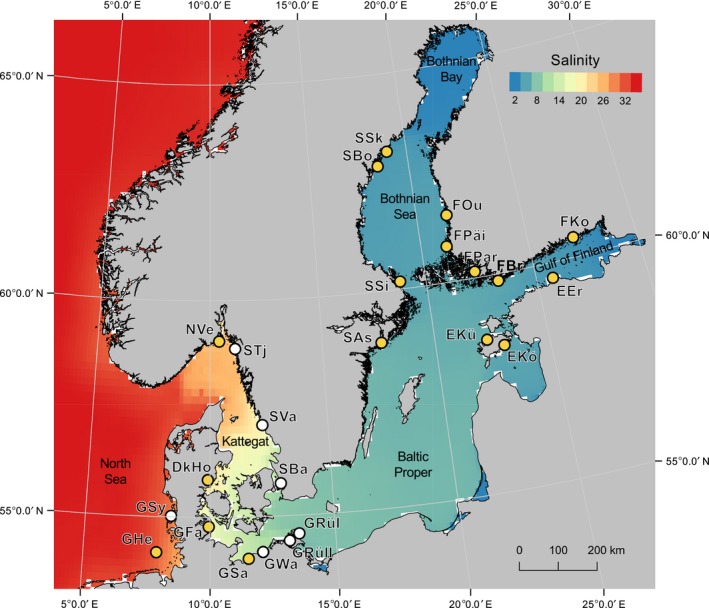
Map showing the location of the 24 sampling stations along the salinity gradient between the North and Baltic seas, where *Fucus vesiculosus* individuals were collected. Yellow circles indicate stations where morphological traits were measured (see details in the main text). GHe: Helgoland, GSy: Sylt, NVe: Verdens Ende, STj: Tjärnö, DkHo: Horsens, SVa: Varberg, GFa: Falshöft, GSa: Salzhaff, SBa: Barsebäck, GWa: Warnemünde, GRüII: Rügen West, GRüI: Rügen East, EKü: Küdema, FPar: Paraistenportti, FBr: Brännskär, SAs: Askö, FPäi: Päiuäkarit, EEr: Eru, EKo: Kõiguste, FOu: Outoori, SSi: Singö, SBo: Bönhamn, SSk: Skagsudde, FKo: Kotka. See the main text for details on the salinity data used in the map

All *F. vesiculosus* individuals were collected in September 2011. The sampling was done simultaneously in 24 stations, four from the North Sea and 20 covering the major sub‐basins of the Baltic Sea where *F. vesiculosus* is present (Figure 2). The simultaneous collection was meant to minimize differences in the physiological stage of individuals caused by temporal changes in environmental conditions (particularly temperature and light). At each station, an area near the upper limit of the *F. vesiculosus* stand, preferably between 0.5 and 1.0 m depth, was chosen. In the Bothnian Sea (Figure 2), the sampling was performed between 0.5–3.5 m, due to ice‐scouring of the shallower growing thalli. Ten individuals representative of the local population with regard to size and fouling coverage were collected 5 m apart from each other to reduce the probability of collecting siblings (Tatarenkov et al., 2007). Each individual was stored in a freezer bag and labeled. All samples were transported to the laboratory in cooler boxes at temperatures below 5°C, then frozen at −20°C within 12 hr, and shipped to the GEOMAR Helmholtz Centre for Ocean Research Kiel (Germany) on dry ice. Once in Kiel, samples were stored at −20°C until further processing.

### 2.2 Morphological traits

Morphology is (within genetic limits) an integrative response of *F. vesiculosus* to environmental drivers such as irradiance, nutrients, temperature, salinity, and/or hydrology (see Rinne, Björkman, Sjöqvist, Salovius‐Laurén, & Mattila, 2018 and citations therein). The frond length, frond width, and stipe width were directly measured in the field for every individual collected in 17 of the 24 stations, covering the overall salinity gradient (see Figure 2). The frond length was measured from the holdfast to the most distal tip with a ruler to the nearest 1 cm. The frond width was measured between the youngest and the next youngest dichotomy. The stipe width was taken between the holdfast and the oldest dichotomy. Both frond and stipe width were measured with a caliper to the nearest 1 mm. The number of fronds from each single holdfast was counted (see Bergström, Tatarenkov, Johannesson, Jönsson, & Kautsky, 2005 for further details).

### 2.3 Biochemical traits

#### 2.3.1 Pigment analysis

While surface concentrations of chlorophyll *a* and fucoxanthin can be considered as rough proxies of the epiphyte load (Saha, Rempt, Grosser, Pohnert, & Weinberger, 2011), tissue concentrations indicate the light‐harvesting ability of *F. vesiculosus*. Most of the pigment content in surface extracts is contributed by micro‐ and macroepiphytes (Saha et al., 2011), and the increase in surface chlorophyll *a* and fucoxanthin is correlated with the increase of autotrophic epibiota. Chlorophyll *a* and fucoxanthin were quantified in tissue and surface extracts by high‐performance liquid chromatography (HPLC). Tissue extracts were generated by overnight incubation of 50 mg freeze‐dried and powdered *F. vesiculosus* in 2 ml isopropanol on a shaker at 4°C. Surface extracts were generated by dipping *F. vesiculosus* for 5 s into a mixture of methanol and hexane (1/1, v/v), dried in vacuo, and taken up in 2 ml isopropanol. The dipping technique can extract surface compounds without damaging the epidermis of *F. vesiculosus* (see Saha et al., 2011). Further dilution in isopropanol was conducted with tissue or surface extracts, if they were too concentrated for direct HPLC analysis. Of each extract, 200 µl was filtered through 0.20 µm syringe filters and analyzed using a liquid chromatography (Varian 940‐LC; Agilent Technologies, Inc.) with diode array detector (DAD). Following Saha et al. (2011), the extracts were separated on a CC 250/4.6 Nucleodur 100‐5 silica gel column (Macherey Nagel). The injection volume was 10 μl. The LC mobile phases were A = heptane and B = ethyl acetate (HPLC‐grade; Carl Roth). The LC gradient with a flow rate of 2 ml/min was 0% solvent B from 0 to 2.5 min; 15% B at 5 min; 85% B at 7.5 min; 100% B at 12.5 min; and 0% B at 15 min. Absorption at 450 nm was used to detect fucoxanthin, while absorption at 660 nm was used to detect chlorophyll *a* and phaeophytin *a*. Solvent blank samples and pigment standards at different concentrations were repeatedly injected between the samples. Fucoxanthin and chlorophyll *a* standards were purchased at Cayman Chemical and Sigma‐Aldrich, respectively. Phaeophytin *a* standard was produced from chlorophyll *a* standard by acidification with HCl (1 M).

#### 2.3.2 Carbon, nitrogen, and mannitol content

Nitrogen (N, a proxy of total protein in seaweeds) and mannitol (carbohydrate) concentrations are related to the quality of *F. vesiculosus* as food source (Angell, Mata, Nys, & Paul, 2016; Weinberger et al., 2011). In addition, mannitol is a major carbon (C) and energy storage compound of brown algae (Groisillier et al., 2014). A single side branch from each individual was freeze‐dried and ground to powder using a ball mill (Mikro‐Dismembrator U, B. Braun Biotech International GmbH), and three sub‐samples of 2 mg from each individual were loaded and packed into tin cartridges (6 × 6 × 12 mm). These packages were combusted at 950°C and the absolute contents of C and N were automatically quantified in an elemental analyzer (Vario EL III; Elementar Analysensysteme GmbH) using acetanilide as standard according to Verardo, Froelich, and McIntyre (1990). For mannitol measurements, 10–20 mg dry weight were used per sample according to the ethanol‐based extraction method described by Karsten, Thomas, Weykam, Daniel, and Kirst (1991) and the analytical protocol of Nitschke, Boedeker, Karsten, Hepperle, and Eggert (2010) using an HPLC system with isocratic elution equipped with a differential refractive index detector (Agilent Technologies, Inc.).

#### 2.3.3 Quantification of phlorotannins

Phlorotannins are polyphenolic compounds found in brown algae, both in a soluble form and bound in cell walls, involved in defensive functions as well as in cell‐wall hardening (Koivikko, Loponen, Honkanen, & Jormalainen, 2005). Apical tips were sampled, freeze‐dried, powdered, and stored at −20°C until phlorotannin determination. The total phlorotannin content, that is all different phlorotannins pooled, was determined from 10 mg of the sample. Algal powder was weighed into 2 ml tubes and extracted twice with 1,400 µl of acetone and water (80/20, v/v). During extraction, the tubes were first shaken on a vortex mixer for 5 min, then incubated over night at 4°C, shaken with a planary shaker (280 min^−1^) for 3 hr, and finally centrifuged to separate solvent and residue. Solvent fractions obtained after extractions were pooled. Acetone was evaporated in a concentrator and water was evaporated in a freeze‐dryer. Extracts were dissolved in 1 ml of UPLC‐grade water and filtered. Samples were then analyzed using a modified Folin–Ciocalteu method with gallic acid as standard, as described in Salminen and Karonen (2011). The absorbance of the samples at 730 nm was measured in a Multiskan Ascent reader (Thermo Electron Corporation) and calculated as the average absorbance of three measurements per sample.

### 2.4 Palatability assays

The palatability of *F. vesiculosus* specimens for the Pan‐Baltic isopod grazer *Idotea balthica* was compared in preference feeding assays. *I. balthica* is a major grazer in littoral environments of the Baltic Sea and the main consumer of *F. vesiculosus* along the entire salinity gradient (e.g., Jormalainen, Honkanen, & Heikkilä, 2001; Schaffelke, Evers, & Walhorn, 1995). Assays with algal material from all sampled stations were performed in Kiel (Germany), using *I. balthica* from the Kiel Fjord. Food pellets containing mosquito mesh (mesh size 1 mm) were prepared from algal powder and agar as described in Weinberger et al. (2011) (see in this study a detailed discussion of the advantages of using reconstituted food pellets in palatability assays). Two pellets containing *F. vesiculosus* powder, one from a sampled individual and another from reference algal material (collected at Hubertsberg, Germany, in February 2006), and a single *I. balthica* were placed into a Petri dish with 30 ml of seawater. Petri dishes were incubated for up to 48 hr in darkness (*I. balthica* is more active under dark conditions) and at 15°C (mean temperature of the Kiel Fjord in spring‐summer—15.0 ± 3.6°C—and at which initial grazer cultures were acclimated). The incubation of the Petri dishes was stopped individually when 25%–75% of at least one of the two pellets had been eaten. The amount of material that was eaten from the sample and reference pellet was quantified by counting the number of mesh squares emptied by a grazer. Numbers of emptied mesh squares counted in 3 sub‐replicates for each sampled *F. vesiculosus* (and the respective reference pellet) were averaged and evaluated as logarithmic odds ratios as described in Weinberger et al. (2011). In order to evaluate potential confounding effects related to the origin of the used grazers, palatability assays were repeated using selected samples (*F. vesiculosus* from Kotka, Rügen East and Tjärnö, Figure 2) and *I. balthica* from different populations. These assays were conducted at Bremerhaven (Germany) using grazers from Helgoland (Germany), at Kiel (Germany) using grazers from the Kiel Fjord and at Turku (Finland) using grazers from the Archipelago Sea (Finland).

### 2.5 Environmental predictors

Only variables that were either reported or expected to have significant ecological impacts on *F. vesiculosus* were included in the analysis. Whenever possible, observational data were used instead of modeled values. Measured data were obtained from the existing national monitoring programs. When measured values for single sites and points in time were missing, relationships between actual and modeled values were used to derive estimates for the missing data. For some variables we were only able to use modeled values (see further details below).

Total nitrogen and phosphorus concentrations (from now on referred to as nitrogen and phosphorus) were used as a proxy for nutrient availability and enrichment (HELCOM, 2018). Only winter values, measured at each station for the period 2010–2011, were used since they best represent the regional nutrient availability in the Baltic Sea before being assimilated, and consequently reduced, by pelagic and benthic primary producers during spring, summer, and autumn (HELCOM, 2018). *F. vesiculosus*, as other perennial brown algae species, accumulates nitrogen reserves in winter that it uses in the following seasons to grow under depleted nutrient conditions (Lehvo, Bäck, & Kiirikki, 2001).

Modeled temperature and salinity data were produced by the Swedish Meteorological and Hydrological Institute (SMHI) using the echam5/RCAO model, covering the whole Baltic Sea at a grid resolution of 2 NM (Meier et al., 2012). These data were supplemented by modeled temperature and salinity values for the North Sea and surrounding regions on a 0.25° × 0.5° latitude–longitude grid (Bersch, Gouretski, Sadikni, & Hinrichs, 2013). The mean annual salinity, mean annual temperature, and mean summer temperature (from now on referred to as salinity, annual temperature, and summer temperature) were obtained from these hydrodynamic model calculations over the period 2010–2011. The Simplified Wave Model method was used to calculate the wave exposure for mean wind conditions during 2010–2011. In order to assure a balanced representation of exposure and wind in the fetch model, distance to shore was expressed in km and wind conditions in m/s (modified from Isæus, 2004). The STRÅNG model system (Landelius, Josefsson, & Persson, 2001) was used to obtain mean annual fields of photosynthetically active radiation (PAR, μmol s^−1^ m^−2^) at a horizontal resolution of 11 × 11 km over the period 2010–2011 (http://strang.smhi.se/).

### 2.6 Statistical modeling

Before modeling the observed changes in *F. vesiculosus* traits, the collinearity between the explanatory variables was tested by calculating Spearman's rank correlation coefficients (Figure S1). In those cases where the correlation between two variables was >.50 and significant, one of them was removed from the set of predictors used in the modeling processes. The final decision for removing a variable was based on (a) prior knowledge on its relevance for explaining ecological and/or physiological responses of the species, (b) the number of high and significant correlations with other predictors (variables strongly correlated with multiple other predictors were preferentially removed) and (c) the existence of a second predictor of similar nature that could account for the variation explained by the one removed. From the seven variables initially considered (salinity, annual temperature, summer temperature, wave exposure, PAR, and nitrogen and phosphorus concentrations), annual temperature and phosphorus were not included in the models. Annual temperature strongly correlated with salinity, summer temperature, and wave exposure (Figure S1). Thus, only summer temperature represented the thermal environment in the adjusted models, since most of the variation potentially explained by annual temperature was covered by this variable. In addition, summer temperature exhibited a weak correlation with other predictors. Phosphorus was positively correlated with nitrogen and summer temperature. Thus, just nitrogen was included in the modeling process to represent nutrient availability and enrichment.

Generalized additive mixed models (GAMM) were used to determine the relative effect and contribution of environmental variables in explaining observed changes in the traits of *F. vesiculosus* (see a summary of analyzed traits in Table 1). GAMM were chosen as modeling tool due to their flexibility in adjusting nonlinear trends, the increase in model complexity without compromising the interpretability of obtained results and the well‐established set of associated tools for model simplification and comparison (Burnham & Anderson, 2002; Guisan, Edwards, & Hastie, 2002; Venables & Ripley, 2002). GAMM were fitted in R using the gamm4 package (Wood & Scheipl, 2017) through the wrapper function uGamm from the package MuMIn (Bartoń, 2018). uGamm allowed the usage of the function dredge for the automatic generation of models for all potential combinations of explanatory variables (see details below). The modeling process of every trait started by setting a global model, which included all environmental variables (retained after testing for co‐linearity) as fixed effects. The variables were included as smooth terms using penalized cubic regression splines. The maximum number of degrees of freedom of each smooth term was limited to 3 to avoid overfitting. The identity of the sampling stations was included as a random intercept in order to (a) deal with the nonindependence of the data, since all *F. vesiculosus* individuals measured in each sampling station were included in the analysis to consider the interindividual variability; (b) account for local sources of variability not considered by the included environmental variables; and (c) represent in a simple way the strong genetic population structure that *F. vesiculosus* exhibits even at short geographic distances (Ardehed et al., 2016; Tatarenkov et al., 2007). The Gaussian distribution and identity link function were used for fitting all models. A logarithmic transformation was applied to those traits that exhibited significant departures from normality (Table 1).

**Table 1:** Morphological and biochemical traits of *F. vesiculosus* analyzed in the present study

Trait	Unit	Transformation
Frond length	cm	Ln
Frond width	mm	–
Stipe width	mm	Ln
Number of fronds	–	Ln
Surface fucoxanthin	ng/cm^2^	Ln
Tissue fucoxanthin	µg/g	Ln
Surface chlorophyll a	ng/cm^2^	Ln
Tissue chlorophyll a	µg/g	–
Mannitol	mg/g	Ln
Phlorotannins	mg/g	Ln
C:N ratio	–	Ln
Relative palatability	–	–

Note

The units and applied transformation are indicated for each trait.

Abbreviation: Ln, natural logarithm.

To avoid arbitrary decisions associated with stepwise model selection techniques, all potential candidate models were run using additive combinations of environmental predictors (Mundry & Nunn, 2008). For each trait, 32 models (including global and null models) were automatically generated using the function dredge. An information‐theoretic approach has been followed to determine the relative support in favor of a given model. The Akaike information criterion corrected for small sample sizes (AICc) was calculated for each model. Models for every trait were ranked based on the AICc, and differences in AICc (∆AICc) and AICc weights (AICcw) were determined. AICcw can be interpreted as the relative likelihood of a model to be the best given the overall set of candidate models and the provided data (Wagenmakers & Farrell, 2004). The relative importance of each variable retained in the best model was determined as the sum of AICcw of those models with a ∆AICc ≤4 where the variable was included (Burnham & Anderson, 2002). The marginal *R* ^2^ (i.e., variance explained by fixed effects, m*R* ^2^) and conditional *R* ^2^ (i.e., variance explained by fixed and random effects, c*R* ^2^) for the best GAMM were calculated using the function r.squaredGLMM from the package MuMIn (Nakagawa & Schielzeth, 2013). For this purpose, the best GAMM was translated into a generalized linear mixed model (GLMM) by using the package lme4 (Bates et al., 2018). The adequacy of every final candidate model was evaluated through a detailed analysis of the plots of residuals.

## 3 RESULTS

### 3.1 Morphological traits

The considered environmental variables were relevant in explaining the observed phenotypic variation in two of the four morphological traits analyzed. The modeling process identified a subset of relevant predictors for frond length and number of fronds, while none of the considered environmental variables explained the observed variation in frond and stipe width (Table S1).

**Figure 3:**
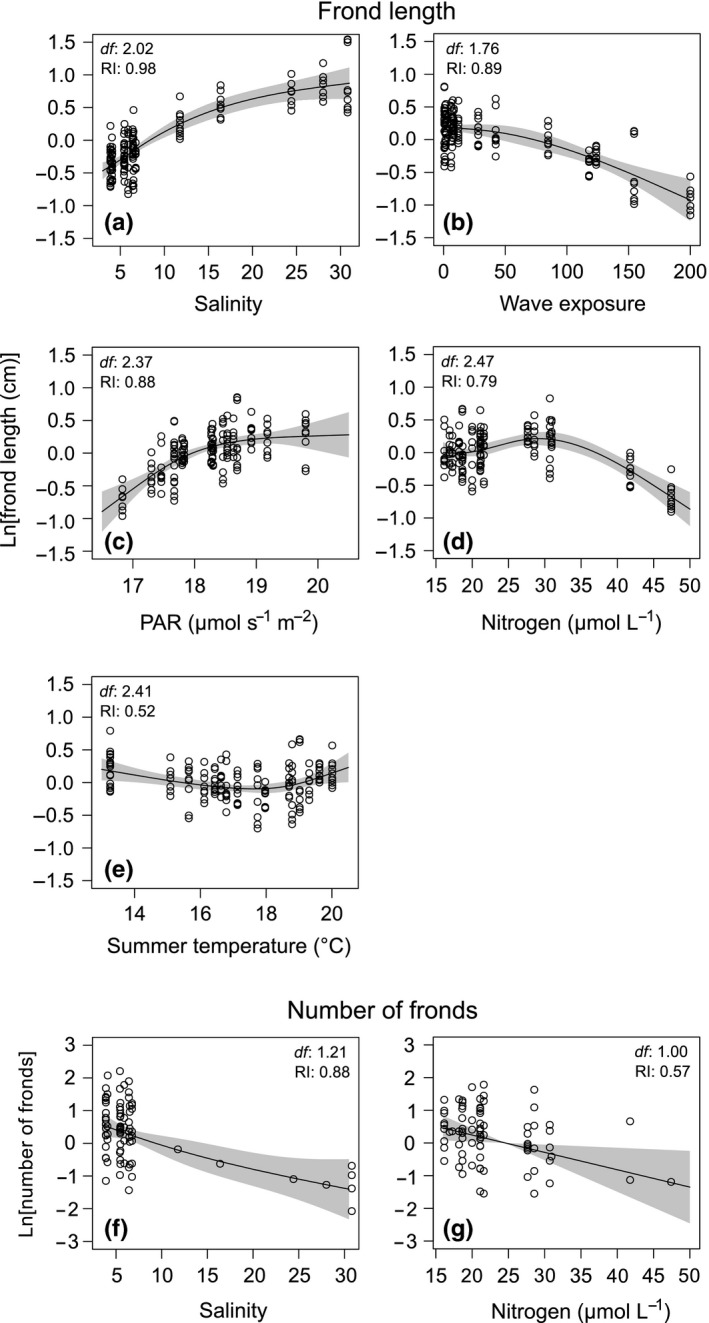
Generalized additive mixed models (GAMM) relating morphological traits of *Fucus vesiculosus* and environmental predictors retained in the applied modeling process (see main text for further details). The *y*‐axes indicate the partial effects of salinity (a), wave exposure (b), photosynthetically active radiation (PAR, c), nitrogen (d) and summer temperature (e) on frond length, and of salinity (f) and nitrogen (g) on the number of fronds. The *y*‐axes are centered and expressed on the scale of the response variable. Gray shaded areas indicate 95% confidence intervals. Estimated effective degrees of freedom (*df*) and the relative importance (RI) are presented for each environmental predictor

In the case of frond length, the model that included all five predictors (salinity, summer temperature, wave exposure, PAR, and nitrogen) was identified as the most parsimonious model (Table S1). Based on the AICcw, this model was 1.67 times more likely than the next best model, which excluded summer temperature (Table S1). The fixed effects (i.e., environmental variables) of the highest ranked model explained 59% of frond length variance (m*R* ^2^). The explained variance was not improved when random effects (i.e., identity of the populations from where *F. vesiculosus* individuals were sampled) were considered (c*R* ^2^, Table 2). Salinity was the most important predictor, being followed in decreasing order by wave exposure, PAR, nitrogen, and summer temperature (Table 2). The frond length increased until a salinity of 15. At higher salinities, the mean frond length of *F. vesiculosus* varied only to a minor extent (Figure 3a). Frond length decreased with wave exposure (Figure 3b). An asymptotic behavior was observed in relation to PAR. The increase in frond length up to 18–19 μmol s^−1^ m^−2^ PAR, reached an upper limit in stations with higher irradiance (Figure 3c). Nitrogen concentration was also retained as a relevant predictor. The observed hump‐shaped trend showed a maximum at 30 µmol/L of nitrogen, value over which the size of the species decreased (Figure 3d). The unexpected nonlinear trend in response to summer temperature (Figure 3e), the least important of the retained variables, might reduce the residual variability improving the overall adjustment of the model.

**Table 2:** Best generalized additive mixed models (GAMM) for morphological, biochemical and palatability traits of *Fucus vesiculosus*

Response	Predictor	*df*	*F*‐value	*p*‐value	RI	m*R* ^2^	c*R* ^2^
Frond length	Salinity	2.02	53.22	<.001	0.98	.59	.59
	Wave exposure	1.76	40.63	<.001	0.89		
	PAR	2.37	23.51	<.001	0.88		
	Nitrogen	2.47	11.67	<.001	0.79		
	Summer temperature	2.41	5.26	<.001	0.52		
Number of fronds	Salinity	1.21	5.94	.016	0.88	.34	.71
	Nitrogen	1.00	10.63	.002	0.57		
Surface fucoxanthin	Salinity	1.00	12.74	<.001	0.91	.16	.40
Tissue fucoxanthin	Salinity	1.00	10.40	.001	0.99	.17	.50
Surface chlorophyll *a*	Salinity	2.27	17.93	<.001	1.00	.44	.44
	PAR	1.00	21.53	<.001	0.93		
	Summer temperature	2.75	19.68	<.001	0.93		
	Nitrogen	1.00	7.18	<.001	0.91		
Tissue chlorophyll *a*	Salinity	1.00	40.61	<.001	1.00	.34	.44
	PAR	1.00	10.37	.002	0.86		
	Wave exposure	1.56	6.37	.022	0.67		
Mannitol	Salinity	1.55	5.72	.005	1.00	.69	.83
	Wave exposure	2.56	22.70	<.001	0.90		
Phlorotannins	Salinity	2.50	9.28	<.001	0.95	.40	.74
C:N ratio	Salinity	1.99	9.25	<.001	0.97	.41	.88
Relative palatability	PAR	1.00	8.171	.005	0.65	.23	.41
	Salinity	1.56	4.29	.014	0.56		
	Wave exposure	1.00	8.242	.005	0.53		

Note

The estimated degrees of freedom (*df*) for the smoother of each environmental variable, their significance (*p*‐value) and the relative importance (RI) are presented. The variance explained by fixed effects (i.e., only environmental predictors) and by both fixed and random effects (i.e., environmental predictors and the identity of the populations from where *F. vesiculosus* individuals were sampled) are indicated by the marginal *R* ^2^ (m*R* ^2^) and conditional *R* ^2^ (c*R* ^2^).

Salinity and nitrogen were identified as the main environmental predictors of the number of fronds (Table S1), with salinity being the most important one (Table 2). Both variables explained 34% of the variance and 71% when *F. vesiculosus* populations were taken into account (Table 2). The number of fronds decreased with both increasing salinity and nitrogen (Figure 3f,g).

### 3.2 Biochemical traits

#### 3.2.1 Pigments

**Figure 4:**
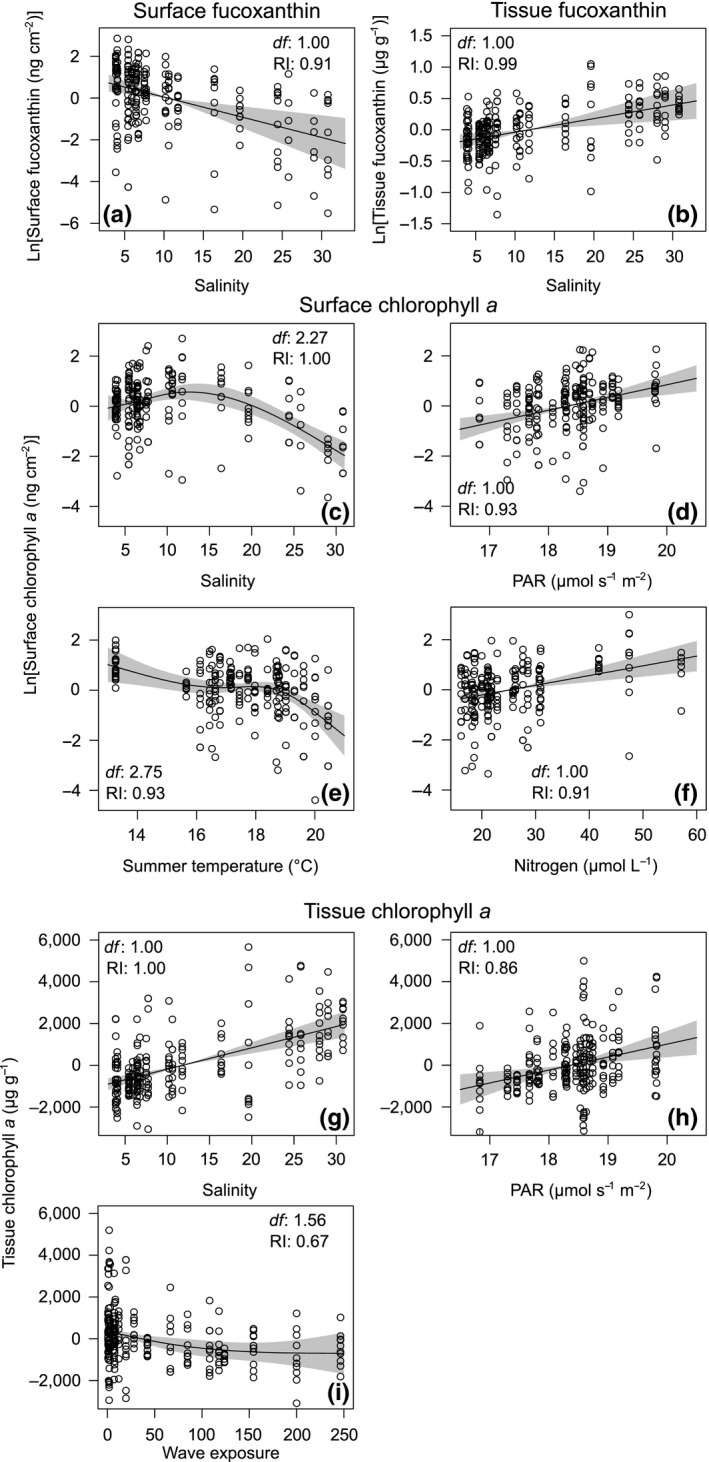
Generalized additive mixed models (GAMM) relating pigment concentrations in tissue and surface extracts of *Fucus vesiculosus* and environmental predictors retained in the applied modeling process (see main text for further details). The *y*‐axes indicate the partial effects of salinity on surface (a) and tissue fucoxanthin (b), salinity (c), photosynthetically active radiation (PAR, d), summer temperature (e) and nitrogen (f) on surface chlorophyll *a*, and salinity (g), PAR (h) and wave exposure (i) on tissue chlorophyll *a*. The *y*‐axes are centered and expressed on the scale of the response variable. Gray shaded areas indicate 95% confidence intervals. Estimated effective degrees of freedom (*df*) and the relative importance (RI) are presented for each environmental predictor

Salinity was the only environmental predictor of the registered spatial changes in surface and tissue fucoxanthin (Table S1). For both surface and tissue extracts, this variable explained about 15% of the variance. The inclusion of the identity of *F. vesiculosus* populations as a random effect increased the explained variance to 40% in the case of surface fucoxanthin and to 50% when tissue samples were analyzed (Table 2). The surface and tissue concentrations of fucoxanthin followed contrasting trends in relation to salinity. The concentration of fucoxanthin in surface samples decreased from brackish to marine stations (Figure 4a). On the contrary, the amount of fucoxanthin in tissue increased with salinity (Figure 4b).

Chlorophyll *a* concentrations were explained by a set of environmental variables in addition to salinity. In decreasing order of importance (Table 2), salinity, PAR, summer temperature, and nitrogen were included in the most parsimonious model obtained for surface chlorophyll *a*. This was the only model retained when a cut‐off threshold of ∆AICc ≤4 was applied (Table S1). No differences were observed between the m*R* ^2^ and c*R* ^2^ (.44), meaning that only the included fixed effects contributed to the explained variance of the model (Table 2). Surface chlorophyll *a* (a proxy of the fouling by autotrophs) showed a hump‐shaped trend with salinity, exhibiting its lowest mean values in North Sea stations (Figure 4c). Surface concentrations increased linearly with PAR and nitrogen and were almost constant along the range of considered summer temperatures, only decreasing in stations with mean values over 19°C (Figure 4d–f). Thus, surface chlorophyll *a* concentrations indicate a higher load of epiphytes in low salinity, nutrient enriched, and high irradiance areas of the Baltic Sea.

Salinity, PAR and wave exposure were identified as the best predictors of tissue chlorophyll *a*. The model that included all three variables was the most parsimonious one according to AICc, and 2.5 times more likely than the model that only included salinity and PAR when AICcw were compared (Table S1). The three variables together explained 34% of the variance and the identity of *F. vesiculosus* populations contributed with an additional 10% (c*R* ^2^ = .44, Table 2). Whereas surface chlorophyll *a* levels showed their lowest values at high salinities, the concentrations of this pigment in tissue increased steadily toward fully marine conditions (Figure 4g). The same trend was found in response to PAR (Figure 4h), while the concentration of chlorophyll *a* in tissue extracts decreased with increasing wave action (Figure 4i).

#### 3.2.2 Mannitol, phlorotannins, and C:N ratio

**Figure 5:**
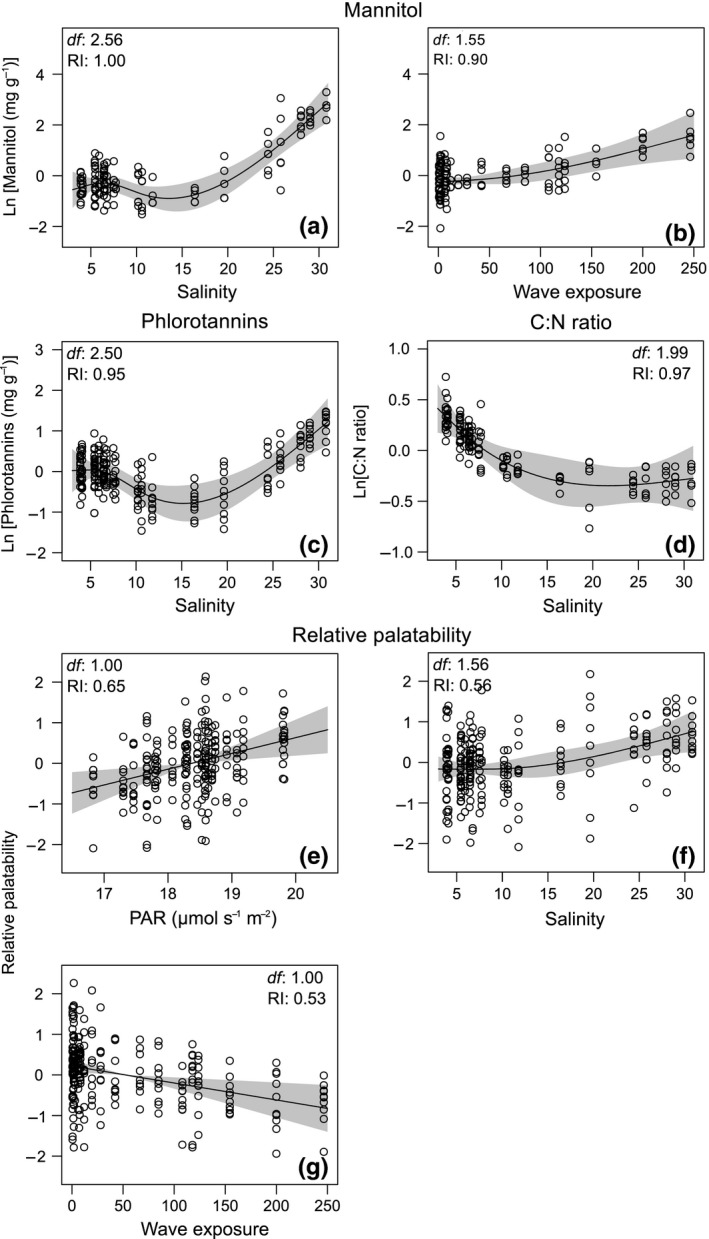
Generalized additive mixed models (GAMM) relating the concentration of mannitol and phlorotannins, C:N ratio and relative palatability of *Fucus vesiculosus* and environmental predictors retained in the applied modeling process (see main text for further details).The *y*‐axes indicate the partial effects of salinity (a) and wave exposure (b) on mannitol, salinity on phlorotannins (c) and C:N ratio (d), and photosynthetically active radiation (PAR, e), salinity (f) and wave exposure (g) on relative palatability. The *y*‐axes are centered and expressed on the scale of the response variable. Gray shaded areas indicate 95% confidence intervals. Estimated effective degrees of freedom (*df*) and the relative importance (RI) are presented for each environmental predictor

Included in the most parsimonious model, salinity and wave exposure explained 69% of the changes in mannitol content (Table 2, Table S1). Differences between algal populations accounted for 14% of the variance (Table 2). The concentration of mannitol stayed constant among stations with salinities up to 10, rapidly increasing in populations of high‐salinity stations (Figure 5a). Populations of *F. vesiculosus* heavily exposed to wave action contained higher concentrations of mannitol (Figure 5b).

According to the AICc and AICcw, the model with the highest empirical support included salinity as the single predictor of the natural logarithm of phlorotannins content and C:N ratio (Table S1). While the response of phlorotannis followed a U‐shaped trend with a minimum at salinities of 15–20 and its highest values around 30, the C:N ratio described a decreasing nonlinear trend along the salinity gradient (Figure 5c,d). This trend was driven by the asymptotic increase in N content with salinity, since C content stayed almost the same among stations (Figure S2).

### 3.3 Palatability assays

When comparing the palatability of algal material of all 24 sampling stations, using *I. balthica* of the same origin (Kiel), the GAMM with the lowest AICc included PAR, salinity, and wave exposure as fixed effects (Table S1). In this model, that had twice as much empirical support as the model with PAR as single environmental predictor (Table S1), the m*R* ^2^ was .23 and the c*R* ^2^ .41 (Table 2). The palatability of *F. vesiculosus* increased linearly with PAR and salinity (Figure 5e,f). In contrast, the increase in wave action decreased the palatability of *F. vesiculosus* for *I. balthica* (Figure 5g).

**Figure 6:**
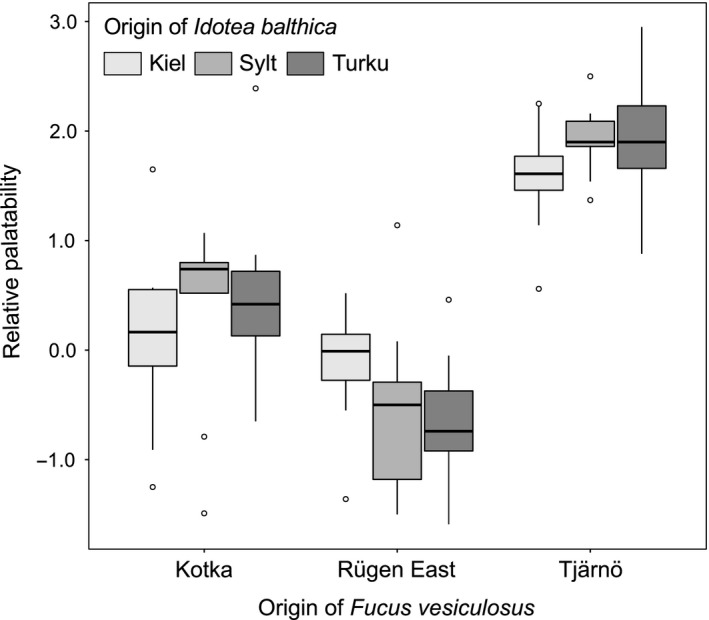
Box‐plots comparing the relative palatability of *Fucus vesiculosus* in preference feeding assays using *Idotea balthica* from different origins. Algal material from Kotka (Finland), Rügen East (Germany) and Tjärnö (Sweden), and grazers from Kiel (Germany), Sylt (Germany) and Turku (Finland) were used. Horizontal solid lines represent the median and boxes the interquartile range (IQR). Whiskers extend to a maximum of 1.5 × IQR and extreme values are represented as open circles beyond the whiskers

The additional palatability assays using *I. balthica* from different locations along the salinity gradient confirm the GAMM results and allow their generalization beyond the origin of the grazer. Differences in palatability were explained by the origin of the algal material, being five times higher in *F. vesiculosus* from Tjärnö (salinity of 25.8) than from Rügen East or Kotka (salinity of 7.5 and 4.1, respectively, Figure 6 and Table S2). These results support the increasing palatability trend in relation to salinity obtained in the modeling process. The origin of *I. balthica* as well as its interaction with the origin of *F. vesiculosus* were not significant (Table S2).

## 4 DISCUSSION

Our study presents an integrative overview of the geographic variation of traits in *F. vesiculosus* and the putative underlying drivers. The general deterioration of traits along the Baltic Sea‐North Sea region was mainly explained by salinity, showing an overall decrease in the performance of *F. vesiculosus* from fully marine to brackish conditions. Although this marine brown alga survived the freshening process that the Baltic Sea underwent in the last 3,000 years (Bäck et al., 1992a, 1992b), the morphology and biochemistry of populations from central and northern basins still exhibit clear signs of osmotic stress. Wave exposure, nitrogen concentration, and light availability, when retained as relevant drivers, had a secondary importance in the best‐fitting models. Nevertheless, the traits' responses to these variables suggest that *F. vesiculosus* is impaired by the impact of waves and eutrophication at sub‐regional and local scales. In addition to the environmental variables considered in the analysis, the geographic origin of algal individuals also contributed to the explained variance of several traits.

The frond length of *F. vesiculosus* was significantly smaller in low‐salinity stations. Nygård and Dring (2008) suggested that the lower growth rate and body size of Baltic *F. vesiculosus* in comparison to those from the Atlantic Ocean is the result of a limited photosynthetic capacity under low‐salinity conditions. They showed that the photosynthesis of *F. vesiculosus* in the Gulf of Bothnia, at a salinity of 5, barely meets the energetic demands required for maintaining positive growth rates. In agreement with these results, the continuous increase in tissue chlorophyll *a* and fucoxanthin with salinity observed in our models suggests a more effective photosynthetic system under more favorable salinity conditions. A previous comparison between populations from the Norwegian and Bothnian seas showed a nearly 4‐fold increase in the concentrations of primary and accessory pigments in high‐salinity individuals with a concomitant increase in their photosynthetic activity (Nygård & Ekelund, 2006). In this context, our large‐scale trends provide additional support for the hypothesis that physiological costs for osmoregulation in low salinities (particularly below 10) affect the photosynthetic system of *F. vesiculosus*, interfering with the energetic balance and development of the alga (Gylle, Nygård, Svan, Pocock, & Ekelund, 2013; Nygård & Dring, 2008; Nygård & Ekelund, 2006). In addition to the impact of low salinity, frond length was negatively affected by wave exposure confirming what previous comparisons between *F. vesiculosus* from exposed and sheltered sites already observed (Bäck, 1993; Bäck et al., 1992a). The decrease in the number of fronds with increasing nitrogen and of frond length at nitrogen concentrations beyond 30 µmol/L show the negative indirect effects of nutrient enrichment on the performance of the *F. vesiculosus*. The increase in water turbidity and decrease in light penetrability, the increase in sedimentation and the favored growth of epiphytic algae are effects of nutrient enrichment that hinder the recruitment and growth of *F. vesiculosus* (Berger, Henriksson, Kautsky, & Malm, 2003; Eriksson & Johansson, 2003; Kautsky, Kautsky, Kautsky, & Waern, 1986). Near fish farms, point sources of nutrient discharges causing local eutrophication, thalli remain smaller (Hemmi et al., 2005). The higher epiphyte load observed in these nutrient enriched areas was stressed as the most likely driver for the reduction in size (Hemmi et al., 2005), causing shading, small scale depletion of nutrients and an increase in grazing pressure (Wahl et al., 2011). As suggested by our models for surface chlorophyll *a* and fucoxanthin, the decrease in salinity might also be accompanied by an increase in epiphytism toward the northern Baltic Sea. This trend could be explained by depressed anti‐fouling capacities of *F. vesiculosus* under osmotic stressful conditions and/or by the increase in bloom forming epiphytic species with brackish affinity (particularly diatoms, Snoeijs, 1994, 1995) toward the northern Baltic Sea.

The decrease in salinity from the North Sea to the Baltic Sea and its impacts on the photosynthetic capacity of *F. vesiculosus* could also explain the observed spatial changes in biochemical composition. The accumulation of carbohydrates such as mannitol (major product of photosynthesis) and laminarin is a key process for C storage in brown algae (see Groisillier et al., 2014; Graiff, Bartsch, Ruth, Wahl, & Karsten, 2015 and citations therein). However, the accumulation of these compounds is expected to occur only when the rate of photosynthesis exceeds the immediate needs of the organism. Thus, the decrease of mannitol in *F. vesiculosus* from marine to brackish stations (up to salinities of 10) could indicate increased energetic requirements, which ‐ combined with an increasingly inefficient photosynthesis ‐ result in the decline of energy reserves. Complementarily, mannitol is an organic solute that plays a major role in the osmoregulation of the Fucales (Bäck et al., 1992b; Wegmann, 1986). Ecophysiological experiments have shown that both marine and brackish populations of *F. vesiculosus* are able to adjust the concentration of mannitol in response to changes in salinity (Bäck et al., 1992b). Therefore, changes in mannitol can also represent an osmotic response.

Salinity was retained as the single environmental predictor of C:N ratio, stressing that the chemical composition of *F. vesiculosus* is affected by its physiological performance in brackish environments. The change in the ratio along the salinity gradient was mainly driven by the variation in N, a proxy for protein content in seaweeds (Angell et al., 2016). In contrast to our results, an early work showed an increase in the concentrations of N based compounds with decreasing salinities (Munda, 1977). N assimilation is tightly related to intermediary respiratory and photosynthetic pathways that provide energy and C skeletons for the production of amino acids. As suggested by Munda (1977), higher respiratory rates of *F. vesiculosus* in low salinity environments result in the consumption of carbohydrate reserves (e.g., mannitol) contributing to the osmoacclimation and favoring the production of amino acids as a secondary effect. However, since the assimilation of N relies on the energy availability of individuals, responses observed in this earlier study to decreasing salinities up to 12 seem not to hold for algae that have grown in conditions close to the osmotic tolerance of the species as observed in our trends.

Our palatability bioassays, which specifically tested for the effects of chemical composition by using reconstituted algal food, suggest that the nutritional value of *F. vesiculosus* increases with light availability and salinity, and decreases with wave exposure. Similar overall trends were observed for the content of tissue chlorophyll *a*. Thus, we hypothesize that the more active photosynthetic system of algae growing under favorable environmental conditions increases the synthesis of carbohydrates and other nutritious compounds, and therefore the palatability of *F. vesiculosus* for herbivores. The increase in mannitol storage and N content with salinity gives further support to this hypothesis. Carbohydrates and mannitol in particular, are used by *I. balthica* and other crustaceans as feeding cues in the assessment of the quality of different food items (Weinberger et al., 2011). The higher concentration of phlorotannins (suggested as anti‐grazing compounds, Koivikko et al., 2005) did not affect the palatability of high‐salinity individuals. This may be due to the inability of our bioassays to reliably detect the influences of highly water‐soluble compounds (Jormalainen, Honkanen, Vesakoski, & Koivikko, 2005) or the less important role of phlorotannins in affecting the palatability of *F. vesiculosus* for *I. balthica*.

The climate‐induced desalination predicted for the end of the century is expected to produce a 30% contraction of the distribution of *F. vesiculosus* in the Baltic Sea (Jonsson et al., 2018; Kotta et al., 2019). The rapid southward and westward expansions of low salinity conditions (with maximum velocities exceeding 100 km per decade) are expected to convert the northern basins (currently at salinities of 4–5) into inhabitable areas for *F. vesiculosus* (Jonsson et al., 2018). However, these predictions based on species distribution models and occurrence data, show only the expected lethal effects of desalination and underestimate the consequences that future nonlethal but highly stressful conditions might have on the performance of *F. vesiculosus* and its interactions with competitors, grazers and epiphytes. In light of our results, the predicted southward reduction of salinity (according to Jonsson et al., 2018) would be followed by a reduction in thallus size, photosynthetic activity and content of nutritious compounds in bladderwrack populations from central and western basins. A decrease in algal size will likely impact the complexity of the habitat provided for invertebrates and fish, the lower photosynthetic activity will suppose a decrease in oxygen production and carbon fixation, and a decrease of palatability will impact the fitness and population growth of herbivores (e.g., *I. balthica*) and their consumers.

Sampling stations accounted for an additional 10%–50% of the variation that remained after adjusting the effects of the considered environmental variables. This suggests that other environmental drivers of local relevance (e.g., biological interactions) or genetic differentiation could also contribute to the observed phenotypic variation among populations. Baltic Sea bladderwrack populations are genetically structured at distances of a few kilometers or even meters (Ardehed et al., 2016; Tatarenkov et al., 2007). Notwithstanding the considerable drifting dispersal capacity of adults (see Jonsson et al., 2018; Rothäusler, Corell, & Jormalainen, 2015 for a detailed discussion), gene flow appears low and the genetic differentiation with the generation of population‐specific phenotypic types is highly probable. Far from conclusive, our estimation of the variance explained by the geographic origin of individuals only stresses the necessity for further studies addressing the contribution of the genetic structure of populations (and spatial changes in biological interactions) to the phenotypic variation of *F. vesiculosus*.

In conclusion, the large‐scale gradient of decreasing salinity was the main and sometimes the only environmental determinant of the geographic variation of traits. Further research on the extent to which observed patterns are the result of phenotypic plasticity or genetic differentiation, and how trait changes are related to population growth rates, are needed to generate predictions of the forthcoming phenotypic changes and the persistence *F. vesiculosus* populations under climate change in the Baltic Sea. In light of our results, populations that will be able to tolerate the expected salinity decline are likely to experience changes in morphological and biochemical traits affecting functions such as habitat formation, primary production and food supply. Beyond the role of salinity and the contribution of other environmental drivers of local relevance, the mechanisms behind the observed geographic variation remain unknown for some traits. Further studies that explicitly consider the genetic divergence among populations (and individuals) and spatial changes in biological interactions (e.g., grazing and epiphytism) would be needed to fully understand how phenotypic variation is ultimately generated and maintained in *F. vesiculosus* at geographic scales.

## CONFLICT OF INTEREST

None declared.

## AUTHOR CONTRIBUTIONS

MW conceived the study. FRB performed the statistical analyses and wrote the first complete draft of the manuscript. JK provided the environmental data. FW and VJ performed the chemical measurements. All authors significantly contributed to the design of the study, collection of the samples, discussion of the results and writing of the final manuscript.

## Data Availability

Data used in the present study are available at PANGAEA: https://doi.pangaea.de/10.1594/PANGAEA.899902
