# Supplementary material for: Geographic variation in fitness‐related traits of the bladderwrack Fucus vesiculosus along the Baltic Sea‐North Sea salinity gradient
Source: Ecol Evol. 2019 Jul 23;9(16):9225–38. doi: 10.1002/ece3.5470 (PMC6706220; doi:10.1002/ece3.5470)
Supplement: Supplementary file 1 [file ECE3-9-9225-s001.docx]

APPENDIX
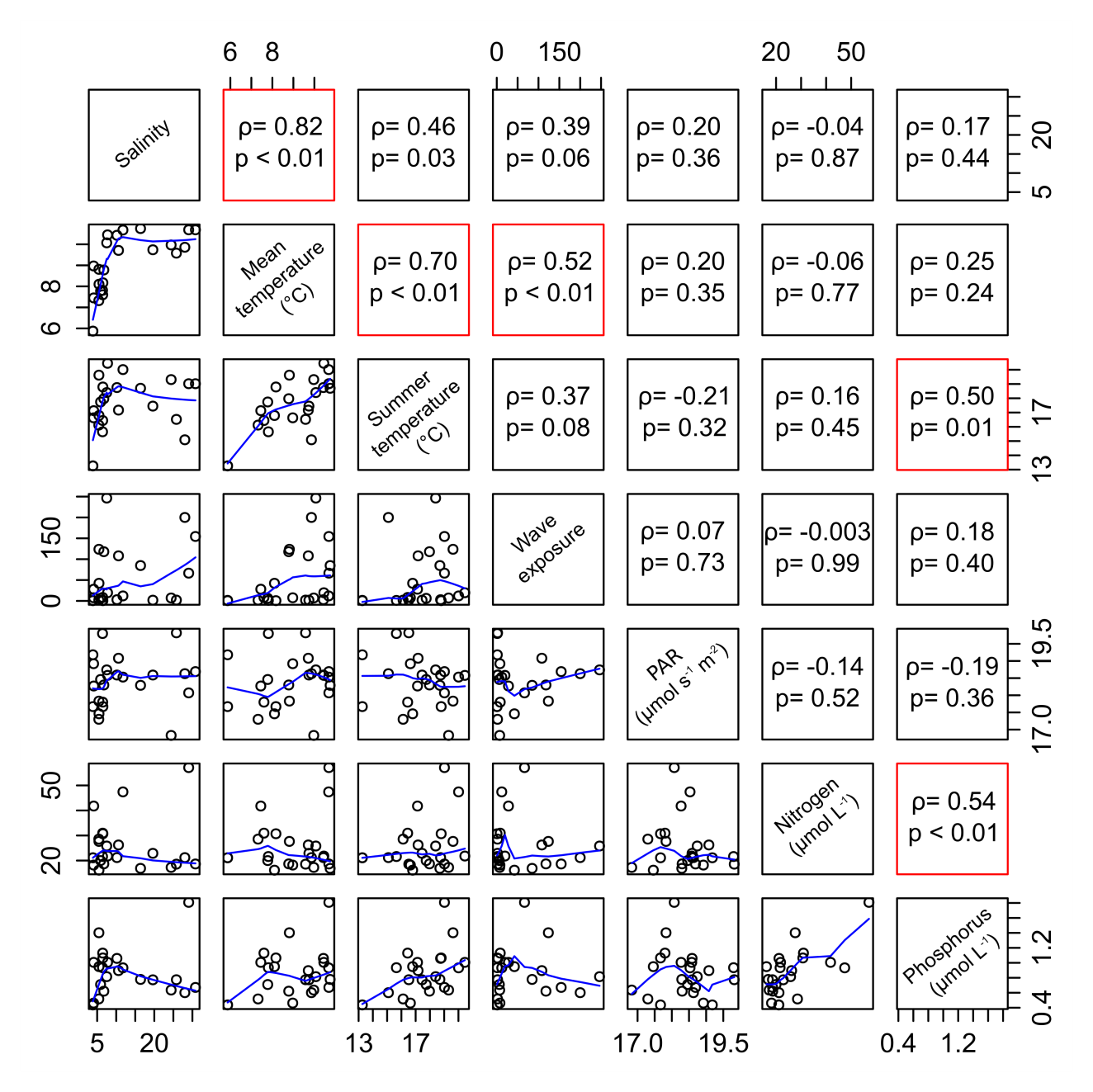


Figure S1. Correlation and scatter plot matrix for the environmental variables considered as potential predictors in the modelling of the traits of *Fucus vesiculosus*. In the upper half of the matrix, Spearman’s rank correlation coefficients (ρ) and the respective p-values are presented. Combinations highlighted in red indicate significant correlations ≥ 0.50. Scatter plots and locally weighted scatter-plot smoother (LOWESS) curves (blue lines) are presented in the lower half of the matrix. PAR: photosynthetically active radiation.

Table S1. Model selection process for the generalized additive mixed models (GAMM) applied in the analysis of the traits of *Fucus vesiculosus*. Environmental predictors included in the models are indicated with crosses. The degrees of freedom (df), log likelihood score (logL), corrected Akaike’s information criterion (AICc), difference in AICc relative to the best model (ΔAICc) and AICc weight (AICcw) are presented for each model. Only models with a ΔAICc ≤ 4 are shown. PAR: photosynthetically active radiation.

| **Trait** | **Environmental predictor** | | | | | **Model performance statistics** | | | | |
| --- | --- | --- | --- | --- | --- | --- | --- | --- | --- | --- |
|  | **Wave**  **exposure** | **PAR** | **Nitrogen** | **Salinity** | **Summer**  **temprature** | **df** | **logL** | **AICc** | **ΔAICc** | **AICcw** |
|  |  |  |  |  |  |  |  |  |  |  |
| Frond length | + | + | + | + | + | 13 | -18.989 | 66.3 | 0.00 | 0.528 |
|  | + | + | + | + |  | 11 | -21.832 | 67.3 | 1.02 | 0.316 |
|  | + | + |  | + |  | 9 | -24.812 | 68.7 | 2.44 | 0.156 |
| Frond width |  |  |  |  |  | 3 | -358.835 | 723.8 | 0.00 | 0.536 |
|  |  |  |  | + |  | 5 | -357.816 | 726 | 2.18 | 0.18 |
|  |  |  |  |  | + | 5 | -358.118 | 726.6 | 2.79 | 0.133 |
|  | + |  |  |  |  | 5 | -358.674 | 727.7 | 3.90 | 0.076 |
|  |  | + |  |  |  | 5 | -358.704 | 727.8 | 3.96 | 0.074 |
| Stipe width |  |  |  |  |  | 3 | -54.533 | 115.2 | 0.00 | 0.327 |
|  |  |  | + |  |  | 5 | -52.862 | 116.1 | 0.88 | 0.211 |
|  |  | + | + |  |  | 7 | -51.262 | 117.2 | 2.00 | 0.12 |
|  |  | + |  |  |  | 5 | -53.617 | 117.6 | 2.39 | 0.099 |
|  |  | + | + | + |  | 9 | -49.474 | 118.1 | 2.86 | 0.078 |
|  | + |  |  |  |  | 5 | -54.079 | 118.5 | 3.31 | 0.062 |
|  |  |  |  |  | + | 5 | -54.166 | 118.7 | 3.49 | 0.057 |
|  |  | + | + |  | + | 9 | -50.022 | 119.2 | 3.96 | 0.045 |
| Number of fronds |  |  | + | + |  | 7 | -188.644 | 392 | 0.00 | 0.181 |
|  | + |  | + | + |  | 9 | -186.579 | 392.3 | 0.30 | 0.155 |
|  |  |  |  | + |  | 5 | -191.078 | 392.5 | 0.54 | 0.138 |
|  | + |  | + | + | + | 11 | -184.692 | 393.1 | 1.08 | 0.106 |
|  | + |  |  | + |  | 7 | -189.24 | 393.2 | 1.19 | 0.1 |
|  | + | + |  | + |  | 9 | -187.366 | 393.9 | 1.88 | 0.071 |
|  |  |  |  |  |  | 3 | -193.859 | 393.9 | 1.88 | 0.071 |
|  | + | + | + | + | + | 13 | -182.866 | 394.1 | 2.09 | 0.064 |
|  |  |  | + | + | + | 9 | -187.634 | 394.4 | 2.41 | 0.054 |
|  | + | + | + | + |  | 11 | -185.821 | 395.3 | 3.33 | 0.034 |
|  | + | + |  | + | + | 11 | -186.062 | 395.8 | 3.82 | 0.027 |
| Surface fucoxanthin |  |  |  | + |  | 5 | -410.363 | 831 | 0.00 | 0.671 |
|  |  | + |  | + |  | 7 | -408.951 | 832.4 | 1.43 | 0.329 |
| Tissue fucoxanthin |  |  |  | + |  | 5 | -1781.117 | 3572.5 | 0.00 | 0.243 |
|  | + |  |  | + |  | 7 | -1779.019 | 3572.5 | 0.03 | 0.239 |
|  | + | + |  | + |  | 9 | -1777.355 | 3573.5 | 1.01 | 0.147 |
|  |  |  |  | + | + | 7 | -1779.712 | 3573.9 | 1.42 | 0.12 |
|  |  | + |  | + |  | 7 | -1779.717 | 3573.9 | 1.43 | 0.119 |
|  | + |  |  | + | + | 9 | -1778.223 | 3575.2 | 2.74 | 0.062 |
|  | + |  | + | + |  | 9 | -1778.777 | 3576.3 | 3.85 | 0.035 |
|  |  |  | + | + |  | 7 | -1780.973 | 3576.4 | 3.94 | 0.034 |
| Surface chlorophyll *a* |  | + | + | + | + | 11 | -313.554 | 650.4 | 0.00 | 1 |
| Tissue chlorophyll *a* | + | + |  | + |  | 9 | -2050.585 | 4120 | 0.00 | 0.473 |
|  |  | + |  | + |  | 7 | -2053.611 | 4121.7 | 1.75 | 0.197 |
|  | + | + |  | + | + | 11 | -2049.313 | 4121.8 | 1.83 | 0.189 |
|  |  | + |  | + | + | 9 | -2052.451 | 4123.7 | 3.73 | 0.073 |
|  | + | + | + | + |  | 11 | -2050.328 | 4123.8 | 3.86 | 0.068 |
| Mannitol | + |  |  | + |  | 7 | -149.713 | 314.3 | 0.00 | 0.482 |
|  | + |  |  | + | + | 9 | -148.095 | 315.6 | 1.29 | 0.253 |
|  | + | + |  | + | + | 11 | -146.154 | 316.3 | 2.08 | 0.171 |
|  | + | + |  | + |  | 9 | -149.076 | 317.5 | 3.25 | 0.095 |
| Phlorotannins |  |  |  | + |  | 5 | -142.26 | 294.8 | 0.00 | 0.591 |
|  |  |  | + | + |  | 7 | -141.418 | 297.3 | 2.56 | 0.165 |
|  |  |  |  | + | + | 7 | -141.559 | 297.6 | 2.84 | 0.143 |
|  | + |  |  |  | + | 7 | -141.909 | 298.3 | 3.54 | 0.101 |
| Carbon:Nitrogen ratio |  |  |  | + |  | 5 | 42.298 | -74.2 | 0.00 | 0.694 |
|  |  |  | + | + |  | 7 | 43.674 | -72.5 | 1.64 | 0.306 |
| Relative palatability | + | + |  | + |  | 9 | -275.943 | 570.7 | 0.00 | 0.323 |
|  |  | + |  |  |  | 5 | -280.921 | 572.1 | 1.42 | 0.158 |
|  |  |  |  |  |  | 3 | -283.193 | 572.5 | 1.81 | 0.13 |
|  | + |  |  | + |  | 7 | -279.324 | 573.1 | 2.46 | 0.094 |
|  |  | + |  | + |  | 7 | -279.394 | 573.3 | 2.60 | 0.088 |
|  | + | + |  |  |  | 7 | -279.454 | 573.4 | 2.72 | 0.083 |
|  |  |  |  | + |  | 5 | -281.607 | 573.5 | 2.80 | 0.08 |
|  | + | + |  | + | + | 11 | -275.747 | 574.7 | 3.99 | 0.044 |


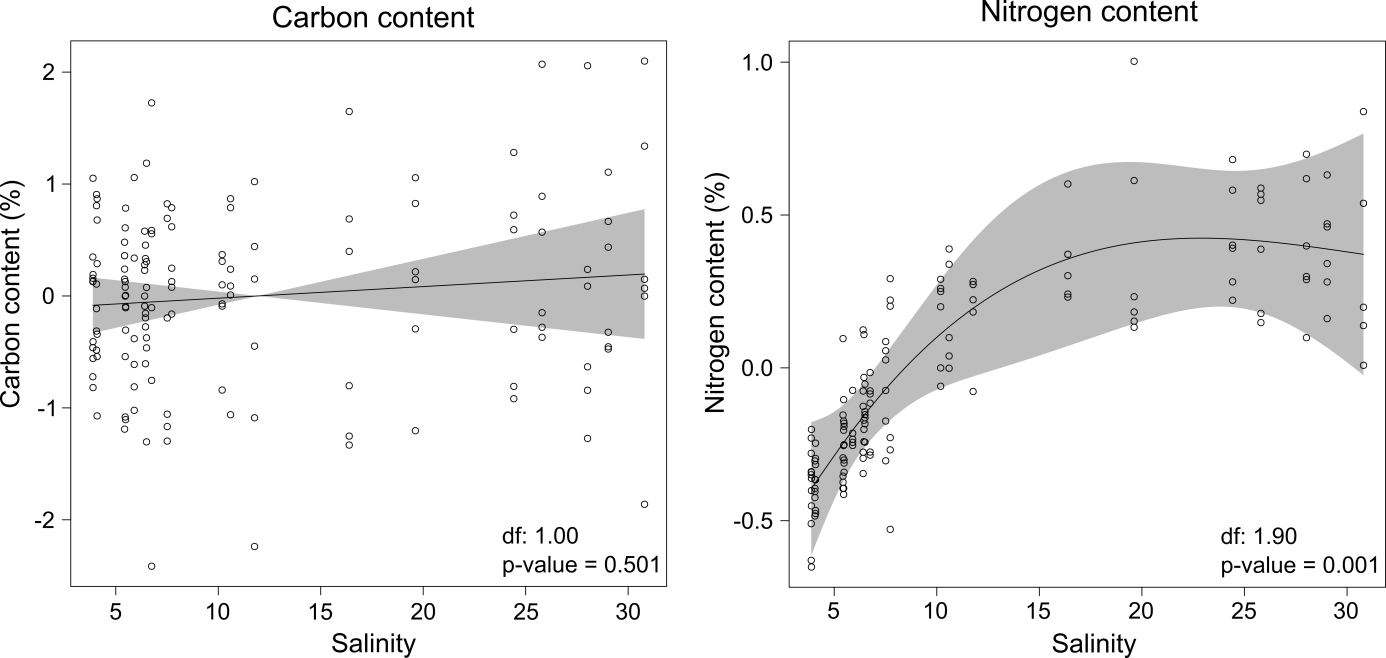


Figure S2. Generalized additive mixed models (GAMM) showing the effects of salinity on the content of Carbon and Nitrogen in *Fucus vesiculosus*. The y-axes indicate the effects of salinity are shown as solid lines. The 95% confidence intervals are indicated as grey shaded areas. The y-axes are centered and expressed on the scale of the response variable. Estimated effective degrees of freedom (df) and p-values are presented for each model.

Table S2. Two-way analysis of variance (Two-way ANOVA, a) and post-hoc test (TukeyHSD, b) for the palatability assays performed using *Fucus vesiculosus* and *Idotea balthica* from different origins (see details in Figure S6). For the post-hoc test only the origin of *F. vesiculosus* was considered, since the origin of *I. balthica* and the interaction between the origin of algal material and grazers were not significant in the Two-way ANOVA. df: degrees of freedom, SS: sum of squares, MSS: mean sum of squares, SE: standard error.

| **(a) Two-way ANOVA** |  |  |  |  |  |
| --- | --- | --- | --- | --- | --- |
| **Source** | **df** | **SS** | **MSS** | **F-value** | **p-value** |
| Origin of *Idotea balthica* | 2 | 0.04 | 0.02 | 0.05 | 0.956 |
| Origin of *Fucus vesiculosus* | 2 | 72.70 | 36.35 | 78.66 | <0.001 |
| Origin of *Idotea balthica*:Origin of *Fucus vesiculosus* | 4 | 2.90 | 0.72 | 1.57 | 0.192 |
| Residuals | 76 | 35.12 | 0.46 |  |  |
| **(b) TukeyHSD** |  |  |  |  |  |
| **Comparison** | **Estimate** | **SE** | **t-value** | **p-value** | |
| Rügen East - Kotka | -0.776 | 0.179 | -4.33 | <0.001 | |
| Tjärnö - Kotka | 1.463 | 0.184 | 7.96 | <0.001 | |
| Tjärnö - Rügen East | 2.238 | 0.181 | 12.39 | <0.001 | |
